# A novel truncating *FBN1* variant in a family with Marfan syndrome

**DOI:** 10.1038/s41439-026-00355-1

**Published:** 2026-06-12

**Authors:** Yuka Ito, Hiroshi Suzumura, Yuko Tanaka, George Imataka, Shujiro Hayashi, Yoshimasa Kawarai, Hiroko Susaka, Miyuki Muramatsu, Hiromi Yuasa, Wakana Soeda, Sakae Ito, Takahiko Kogai, Takahiro Yamada, Katsuhiko Naruse

**Affiliations:** 1 Department of Genetic Diagnosis and Laboratory Medicine, Dokkyo Medical University, Tochigi, Japan; 2 Department of Pediatrics, Dokkyo Medical University, Tochigi, Japan; 3 Division of Clinical Genetics, Dokkyo Medical University Hospital, Tochigi, Japan; 4 Department of Cancer Genome, Dokkyo Medical University, Tochigi, Japan; 5 Comprehensive Cancer Center, Dokkyo Medical University Hospital, Tochigi, Japan; 6 Department of Dermatology, Dokkyo Medical University, Tochigi, Japan; 7 Department of Obstetrics and Gynecology, Dokkyo Medical University, Tochigi, Japan; 8 Graduate School of Nursing, Dokkyo Medical University, Tochigi, Japan; 9 Department of Hematology and Oncology, Dokkyo Medical University, Tochigi, Japan; 10 Department of Ophthalmology, Dokkyo Medical University, Tochigi, Japan; 11 Division of Clinical Genetics, Hokkaido University Hospital, Hokkaido, Japan

**Keywords:** Genetic testing, Disease genetics

## Abstract

Marfan syndrome is caused by pathogenic variants in *FBN1*. We identified a novel heterozygous frameshift variant in *FBN1* (NM_000138.5: c.6784_6787del, NP_000129.3:p.(Gln2262TrpfsTer28)) in an adult male with severe cardiovascular manifestations. The variant was absent from population databases and fulfilled PVS1 and PM2 criteria. This finding expands the mutational and phenotypic spectrum of *FBN1* and highlights the clinical utility of genetic testing in family-based clinical management of Marfan syndrome.

Marfan syndrome (MFS) is an autosomal dominant connective tissue disorder caused by pathogenic variants in *FBN1*, which encodes fibrillin-1, a key component of extracellular microfibrils^1^. Clinical diagnosis is established according to the revised Ghent criteria, with molecular genetic testing providing confirmatory evidence and enabling familial risk assessment^2,3^. Truncating *FBN1* variants have been associated with cardiovascular involvement, including aortic root dilatation and increased risk of aortic events^4,5^. Here, we report a novel truncating *FBN1* variant in an adult male with clinical features of MFS. Genetic testing provided molecular observation of the clinical diagnosis.

A 33-year-old man presented with progressive exertional dyspnea. His height was 174 cm (+0.4 standard deviation (SD)) and weight was 60 kg. Echocardiography revealed aortic root dilatation, with a sinus of Valsalva diameter of 89 mm (Z score 21.66), and severe aortic regurgitation, necessitating a Bentall procedure. Physical examination showed a slender build, pectus excavatum, scoliosis, and a positive thumb sign (Fig. 1B–D). Ophthalmologic evaluation demonstrated myopia without ectopia lentis. These findings were suggestive of MFS based on the revised Ghent criteria ^2^. His mother (height 170 cm (+2.3 SD)) died suddenly at 37 years of age from presumed cardiac causes. A three-generation pedigree is shown in Fig. 1A. Individual III-2 was a stillbirth at 39 weeks of gestation (cause unknown).

**Fig. 1:**
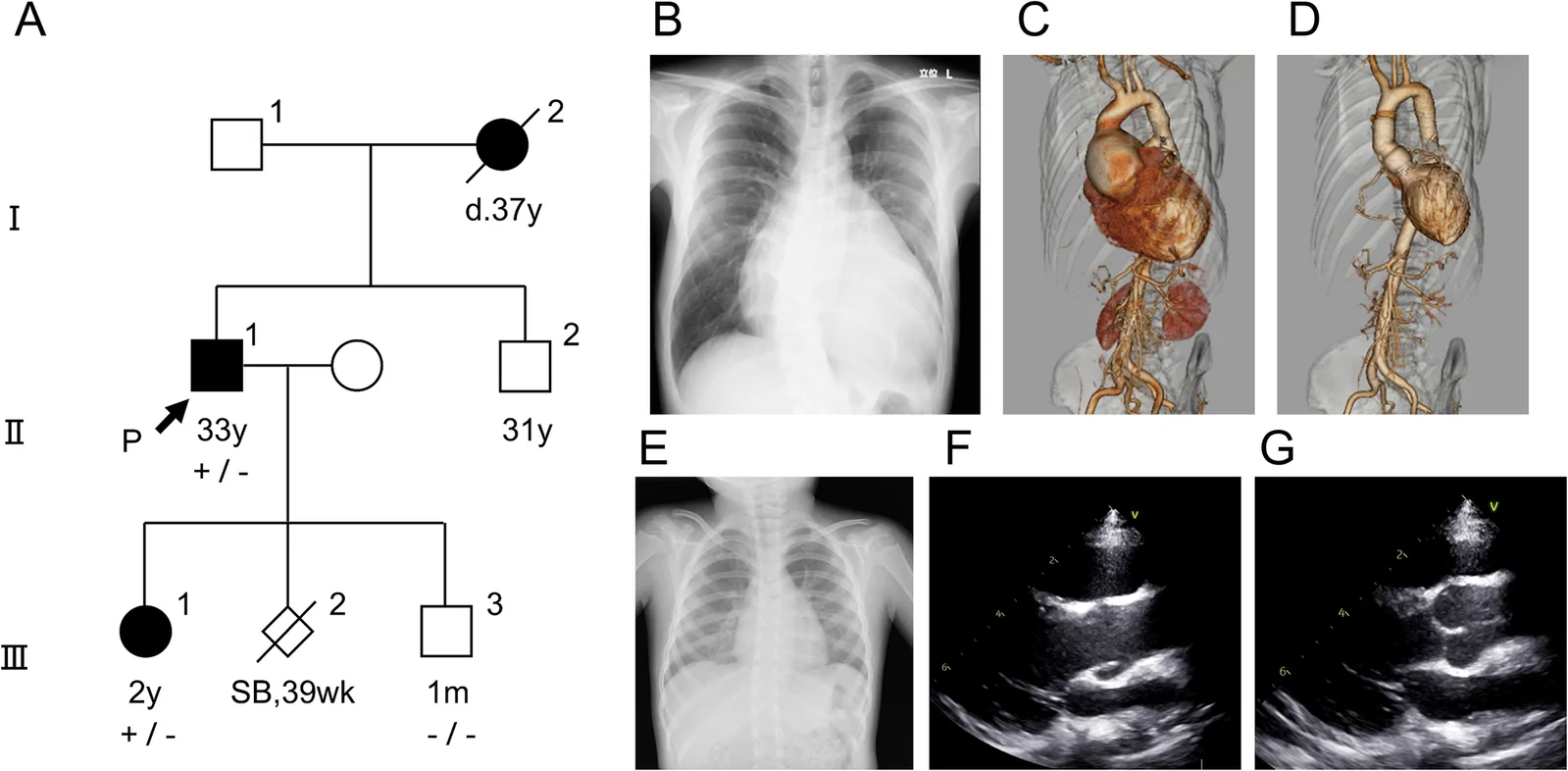
Pedigree, clinical findings, and imaging findings of the family. **A** Pedigree of the family. Filled symbols indicate clinically affected individuals. “d” denotes age at death. Square, male; circle, female; diamond, unspecified sex; slash, deceased; arrow, proband; SB, stillbirth at 39 weeks. Genotypes are shown below each symbol (“+/−”, heterozygous carrier; “−/−”, non-carrier). **B–G** Clinical and imaging findings of affected family members. **B–D** Proband, individual II-1. **B** Chest radiograph of aortic root dilatation and scoliosis. Preoperative (part **C**) and postoperative (part **D**) 3D CT of an aortic root aneurysm. **E–G** Individual III-1. **E** Chest radiograph. **F**, **G** Transthoracic echocardiography during diastole and systole, showing sinus of Valsalva dilatation.

Targeted next-generation sequencing was performed at the Kazusa DNA Research Institute (Chiba, Japan) using a hybrid-capture gene panel, which identified a novel heterozygous frameshift variant in *FBN1* (NM_000138.5: c.6784_6787del, NP_000129.3:p.(Gln2262TrpfsTer28)). The variant is located in exon 56 and introduces a premature termination codon more than 50 bp upstream of the final exon–exon junction, predicting nonsense-mediated mRNA decay. The position of the variant and the predicted molecular consequence are illustrated in Fig. 2. The variant is absent from population databases, including gnomAD (v4.0, GRCh38), and has not been previously reported. According to ACMG/AMP guidelines^6^, the variant fulfills PVS1 (predicted loss of function in a gene where haploinsufficiency is an established disease mechanism) and PM2 (absence from controls). On the basis of the available evidence, the variant was classified as likely pathogenic.

**Fig. 2:**
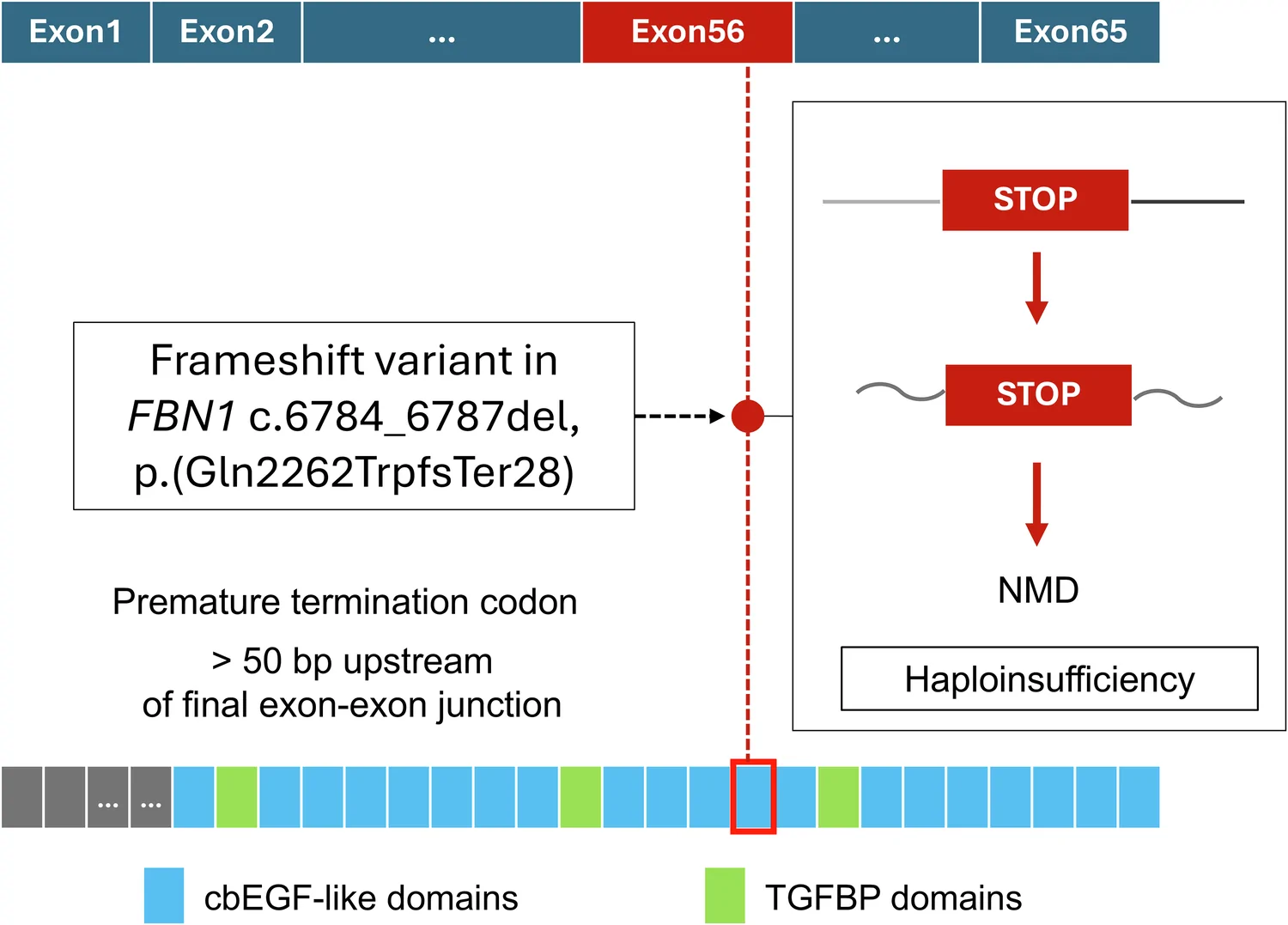
Schematic representation of the *FBN1* frameshift variant. The variant is located in exon 56 and maps to cbEGF-like domain 39. cbEGF calcium-binding epidermal growth factor, NMD nonsense-mediated mRNA decay, TGFBP transforming growth factor beta-binding protein-like.

After identification of the *FBN1* variant in the proband, cascade genetic testing was offered to his two children. The older child, a 2-year-old, who had previously been noted to have tall stature (+1.8 SD), was found to carry the familial variant. Subsequent echocardiographic evaluation revealed mild aortic root dilatation (sinus of Valsalva 20 mm, *Z*-score 2.0, Fig. 1E–G), and the child is currently under cardiologic surveillance. The younger child, 1-month-old, tested negative for the variant and showed no clinical features suggestive of MFS. The familial distribution of the variant was compatible with autosomal dominant inheritance.

Haploinsufficiency due to *FBN1* loss-of-function variants represents a well-established disease mechanism in MFS^1,5^. The present truncating variant is predicted to introduce a premature termination codon leading to nonsense-mediated mRNA decay, resulting in haploinsufficiency. Truncating variants associated with haploinsufficiency have been linked to an increased risk and earlier onset of aortic complications, likely through reduced fibrillin-1 production^4,5,7–9^. Thus, the predicted haploinsufficiency mechanism may contribute to the observed aortic phenotype in this family. Previous studies have also demonstrated altered biomechanical properties of the aorta in MFS, underscoring the importance of careful cardiovascular surveillance^10^.

In addition to enhancing our understanding of its molecular background and associated phenotype, genetic testing contributed to clinical management in this family. Identification of the familial variant enabled cascade testing of the proband’s children, leading to the detection of an affected child and the initiation of regular cardiologic surveillance at an early stage. Conversely, the absence of the variant in the younger child obviated the need for ongoing clinical evaluation. These findings illustrate the practical value of genetic testing in guiding family-based clinical management in MFS^11^.

In conclusion, we describe a novel truncating *FBN1* variant associated with MFS. This report adds to the spectrum of *FBN1* variants and underscores the clinical utility of molecular genetic testing for diagnosis and familial risk stratification in MFS.

## HGV Database

The relevant data from this Data Report are hosted at the Human Genome Variation Database at https://doi.org/10.6084/m9.figshare.hgv.3669.
